# Case report: Improvement of chronic mania after Steven-Johnson syndrome

**DOI:** 10.1192/j.eurpsy.2023.1453

**Published:** 2023-07-19

**Authors:** C. Pardo González, A. Tellez Gomez, C. Sanjuán Ortiz, J. Ribes Cuenca, D. M. Beltrán Cristancho, G. Ribes Jordán

**Affiliations:** Psychiatry, Hospital Universitario i Politécnico La Fe, Valencia, Spain

## Abstract

**Introduction:**

Stevens-Johnson Syndrome is a rare life-threatening condition characterized by severe mucocutaneous epidermal necrolysis and detachment of the epidermis. The condition centers around a delayed-type hypersensitivity reaction with a complex etiology stemming from a variety of causes.

**Objectives:**

To present the case of a patient with a diagnosis of intellectual disability, bipolar disorder and epilepsy who, 14 days after starting treatment with Cariprazine, presented with pseudovesicular skin lesions suggestive of Steven-Johnson syndrome.

**Methods:**

A non-systematic literature review on PubMed database on Steven-Johnson syndrome and other autoimmune processes in patients with bipolar disorder, and the impact on the affective symptoms of the former, was conducted. The clinical case report was prepared through the review of clinical records of the patient.

**Results:**

The authors present the case of a 50-year-old woman, undergoing psychiatric follow-up for more than 30 years with a diagnosis of bipolar disorder. She has a moderate intellectual disability and generalized epilepsy diagnosed at the age of 13. Since the age of 20, the patient has presented clinical manifestations compatible with bipolar disorder.

On a dermatological level, the patient had medical records of hypersensitivity reaction to amoxicillin-clavulanic acid, intolerance to carbamazepine; and toxicoderma and hepatitis after treatment with Lamotrigine, compatible with DRESS syndrome.

At the time of the study, psychopharmacological treatment consisted in valproic acid, lithium and cariprazine (the latter being introduced 14 days earlier). Pseudovesicular and papular skin lesions were observed, with a dianiform appearance and central necrosis.

Prior to the debut of the dermatological condition, the patient showed a decompensation of her bipolar disorder, with escalating irritability, soliloquies, verbosity and hostility towards her parents, with episodes of psychomotor agitation.

After the appearance of the skin lesions, a striking clinical change was observed, with an almost complete remission of affective symptoms, temporally coincident with DRESS syndrome and cariprazine withdrawal.

**Conclusions:**

In recent years, research on autoimmune diseases and their relationship with mental disorders, such as bipolar disorder, schizophrenia and depression, has become increasingly abundant. The conclusions point to the fact that both disorders could be interrelated even at an etiopathogenic level. In this case report, we discuss a patient with a diagnosis of bipolar disorder with an important component of autoimmune response to different drugs, which seems to have influenced the clinical course of the mental illness.

**Disclosure of Interest:**

None Declared

